# Long-term age-stratified outcomes after surgical and transcatheter aortic valve replacement: a Dutch cohort study

**DOI:** 10.1007/s12471-025-01944-5

**Published:** 2025-04-11

**Authors:** Kees van Bergeijk, Stijn Venema, Ad van den Heuvel, Rik van der Werf, Wobbe Bouma, Yvonne Douglas, Niki Medendorp, Marijke Timmermans, Adriaan Voors, Joanna J. Wykrzykowska, Transcatheter Heart valve Interventions and Cardiothoracic Surgery Registration Committee of the Netherlands Heart Registry

**Affiliations:** 1 Department of Cardiology, University Medical Centre Groningen, University of Groningen, Groningen, The Netherlands; 2 Department of Cardiothoracic Surgery, University Medical Centre Groningen, University of Groningen, Groningen, The Netherlands; 3 Netherlands Heart Registry, Utrecht, The Netherlands

**Keywords:** TAVI, SAVR, Comorbidities, Long-term outcomes

## Abstract

**Background:**

While randomised trials have shown that surgical and transcutaneous aortic valve replacement/implantation (SAVR/TAVI) have similar short- to mid-term outcomes, long-term outcome data are scarce. Additionally, no large-scale long-term follow-up data from Dutch databases and TAVI centres have been reported to inform national guidelines.

**Methods:**

We retrospectively analysed baseline characteristics, 5‑year mortality and re-intervention rates of patients undergoing isolated SAVR or TAVI, stratified by age (65–75, 75–80 and > 80 years old) in the Netherlands Heart Registry.

**Results:**

From 2013 through 2021, 7879 SAVR patients (median age: 73.0 years; interquartile range (IQR): 69.0–77.0; 43.7% female) and 14,461 TAVI patients (median age: 81.0 years; IQR: 77.0–84.0; 49.9% female) were treated in the Netherlands. Patients undergoing TAVI more frequently had chronic obstructive pulmonary disease, diabetes, atrial fibrillation, dialysis, poor mobility, previous stroke, unstable angina and recent myocardial infarction compared with SAVR patients. This higher comorbidity rate in TAVI was observed across all age groups. After 5‑year follow-up, mortality rates were higher after TAVI compared with SAVR (35.5% vs 13.0%; *p* < 0.001). This difference decreased with increasing age (*p* for interaction < 0.001). While the aortic re-intervention rate was low in both cohorts, it was higher after SAVR than TAVI (1.9% vs 0.9%; *p* < 0.001).

**Conclusion:**

Demographics of patients undergoing SAVR versus TAVI in the Netherlands differed substantially. TAVI patients were older and had more comorbidities than SAVR patients, across all age groups. Mortality rates were highest after TAVI, while aortic re-intervention was more common after SAVR. These findings reflect differences in baseline patient characteristics and current daily practice in decision-making by the Heart Teams.

**Supplementary Information:**

The online version of this article (10.1007/s12471-025-01944-5) contains supplementary material, which is available to authorized users.

## What’s new?

- Patients who underwent surgical or transcutaneous aortic valve replacement (SAVR/TAVR) in the Netherlands differed substantially in baseline characteristics, even when stratified by age.
- Mortality rates were high after TAVR compared with SAVR, but long-term mortality rates after SAVR clearly increased with age, while this was less pronounced after TAVR.
- Aortic re-intervention rates were low in both cohorts but higher after SAVR. However, both the reason for aortic valve re-intervention and the reason for withholding such a re-intervention were unknown.

## Introduction

Transcatheter aortic valve implantation (TAVI) was originally performed in patients who were not eligible for surgical aortic valve replacement (SAVR), mainly due to high surgical risk. However, mid-term follow-up data from recent randomised trials increasingly support the equipoise of clinical outcomes of SAVR and TAVI in a younger and lower-risk population [1–4]. This is further supported by 2 recent low-surgical-risk trials showing similar or even better haemodynamic parameters in TAVI patients [3, 4]. Nevertheless, the 2021 European Society of Cardiology/European Association for Cardio-Thoracic Surgery Guidelines give a recommendation for TAVI in patients > 75 years or at high surgical risk (STS-PROM/EuroSCORE II > 8%) [5]. In the Netherlands, the criteria of the National Health Care Institute (*Zorginstituut Nederland*) impose an even stricter age limit of 80 years for TAVI [6].

Current data may not be sufficient for international and local guidelines to change their recommendations and recommend TAVI in younger or lower-risk patients. While long-term follow-up data from low-risk trials are eagerly awaited, data from large registries comparing TAVI and SAVR could support their equivalence, but they are scarce. There have been a few nationwide cohort studies reporting on both SAVR and TAVI outcomes, but they did not include long-term follow-up mortality and re-intervention rates and included predominantly patients with older-generation devices [7–9]. In addition, long-term follow-up data of patients in the Netherlands are lacking.

**Fig. 1:**
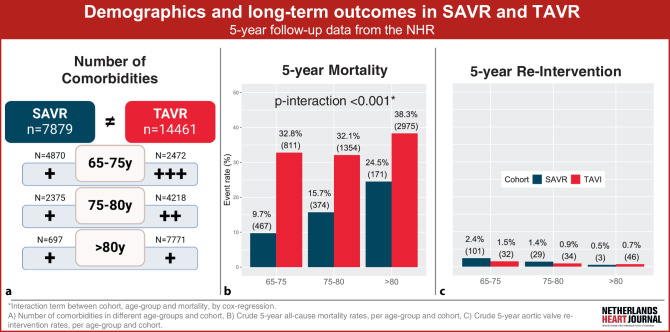
Infographic on demographics and long-term outcomes in SAVR and TAVR

Therefore, we aimed to evaluate real-world data from the Netherlands Heart Registry (NHR) to analyse: (1) baseline characteristics and (2) mortality and aortic valve re-intervention rates at 5‑year follow-up of TAVI and SAVR patients stratified by age (Fig. 1).

## Methods

### Study population and data collection

The NHR is a nationwide quality registry, including data from 15 Dutch heart centres performing TAVI and SAVR, consisting of demographics, clinical characteristics, peri-procedural parameters, complications, mortality status and follow-up data [10]. All patients aged > 64 years receiving isolated SAVR or TAVI in the Netherlands in the period 2013–2021 were eligible for this study. An isolated SAVR or TAVI procedure was defined as a valve replacement that was not combined with any other additional procedure in the same setting, such as coronary artery bypass grafting, left atrial appendage closure or percutaneous coronary intervention in the case of TAVI. All access types for TAVI procedures were included in this analysis. Patients with incomplete follow-up data (e.g. absence of follow-up status or time-to-follow-up) or who had previously undergone SAVR or TAVI were excluded.

### Included parameters

The following baseline data were collected: age, sex, body mass index (BMI) and the EuroSCORE II components (New York Heart Association (NYHA) class III/IV, Canadian Cardiovascular Society class, poor mobility, chronic obstructive pulmonary disease (COPD), diabetes mellitus (defined as insulin use), previous cardiac surgery, active endocarditis, critical pre-operative state, unstable angina, recent myocardial infarction (MI), thoracic aortic surgery and urgency of the operation). Additional parameters included history of atrial fibrillation (AF) and previous stroke and left ventricular ejection fraction (LVEF), based on echocardiographic images, either exact or eyeballing, and pulmonary arterial systolic pressure (PASP), invasively measured or estimated by echocardiography.

### Outcomes

The primary endpoint was all-cause mortality (maximum 5‑year follow-up). The secondary endpoint was aortic valve re-intervention (maximum 5‑year follow-up).

### Statistical analysis

Categorical variables were compared with the chi-squared or Fisher’s exact test (2-sided) and are reported as *n* (%). Continuous variables were compared with the *t*-test or Mann–Whitney U test, and ANOVA or Kruskal–Wallis test for comparison of ≥ 2 groups, and are reported as median (interquartile range; IQR). Because of the known age difference between TAVI and SAVR patients and the different age limits included in the guidelines, the following age groups were defined: 65–75 years, 75–80 years and > 80 years [11].

Because of the known improvements in TAVI technologies and resulting improvements in outcomes, a sensitivity analysis was performed in which patients in the ‘early’ TAVI years (2013–2016) and those in the ‘recent’ years (2017–2021) were compared. In addition, given the higher risk and worse outcomes associated with non-transfemoral access, a sensitivity analysis was performed for only transfemoral TAVI patients. To identify a trend in prevalence of comorbidities across older age groups, *p* for trend was calculated using the Pearson test and Spearman test for normal and non-normal distributed continuous variables, respectively, and the Mantel-Haenszel test for categorical variables.

Due to differences in follow-up time, cumulative crude survival was expressed as *n* (%). Survival analyses were evaluated by a Kaplan-Meier analysis with log-rank tests and Cox proportional hazard modelling. The assumption of proportional hazards was tested by Schoenfeld residuals and the *cox.zph* package test in R, and stratification of the non-proportional hazard was performed if needed. To identify an interaction between older age groups and cohorts for both outcomes, an interaction-term was analysed by Cox regression survival analysis.

All statistical analyses were performed using R software (version 4.3.2.). Statistically significant differences were established at *p* < 0.05.

## Results

### Baseline characteristics

We included 7879 patients who underwent an isolated SAVR procedure and 14,461 patients who underwent an isolated TAVI procedure in the Netherlands in the period 2013–2021. At baseline, SAVR patients were younger than TAVI patients (median age: 73.0 years; IQR: 69.0–77.0 vs 81.0 years; IQR: 77.0–84.0; *p* < 0.001), were less frequently female (3447 (43.7%) vs 7232 (50.0%); *p* < 0.001) and had a higher median BMI (27.3 kg/m^2^; IQR: 24.7–30.4 vs 26.5 kg/m^2^; IQR: 23.9–29.8; *p* < 0.001), a lower NYHA class (class III/IV: 1894 (31.7%) vs 7712 (57.6%); *p* < 0.001) and lower median EuroSCORE II (1.44; IQR: 1.08–2.13 vs 3.25; IQR: 2.01–5.45; *p* < 0.001) (Tab. 1). After dividing patients into age groups (65–75, 75–80 and > 80 years old), there was no statistically significant difference in the distribution of sex by age, but TAVI patients consistently had a higher NYHA class, a higher rate of poor mobility and a higher EuroSCORE II compared with SAVR patients (see Table S1 in Electronic Supplementary Material).

**Table 1:** Baseline characteristics

Variable	SAVR			TAVI			*P*-value
	Total	Missing		Total	Missing		
	(*N* = 7879)	*N*	%	(*N* = 14,461)	*N*	%	
*Demographics*							
Age, years	73.0 [69.0–77.0]	0	0	81.0 [77.0–84.0]	0	0	< 0.001^*^
Female	3447 (43.7)	0	0	7232 (50.0)	0	0	< 0.001^*^
BMI	27.3 [24.7–30.4]	338	4	26.5 [23.9–29.8]	146	1	< 0.001^*^
NYHA class III/IV	1894 (31.7)	1953	24	7712 (57.6)	1078	7	< 0.001^*^
CCS class IV	61 (0.91)	1166	14	302 (2.38)	1807	12	< 0.001^*^
Poor mobility	216 (3.29)	1342	17	1181 (9.89)	2536	17	< 0.001^*^
EuroSCORE II	1.44 [1.08–2.13]	1961	24	3.25 [2.01–5.45]	2681	18	< 0.001^*^
*Comorbidities*							
Chronic lung disease	995 (12.6)	8	0	2818 (19.5)	29	0	< 0.001^*^
Diabetes	1670 (21.4)	96	1	3889 (27.2)	175	1	< 0.001^*^
Atrial fibrillation	565 (11.2)	2886	36	608 (30.2)	12,547	86	< 0.001^*^
Dialysis	22 (0.33)	1141	14	129 (0.90)	189	1	< 0.001^*^
Stroke	360 (4.90)	539	7	1510 (10.5)	15	0	< 0.001^*^
*Cardiac status*							
Unstable angina	11 (0.14)	16	0	44 (0.31)	175	1	0.046^*^
Recent MI	87 (1.11)	8	0	275 (1.92)	102	1	< 0.001^*^
Previous cardiac surgery	302 (3.83)	0	0	2215 (15.5)	219	2	< 0.001^*^
Thoracic aortic surgery	3 (0.04)	0	0	5 (0.04)	306	2	1.000
Endocarditis	212 (2.69)	16	0	2 (0.01)	627	4	< 0.001^*^
Critical pre-operative condition	70 (0.89)	8	0	57 (0.40)	87	1	< 0.001^*^
Urgency	944 (12.5)	362	4	1294 (9.08)	219	2	< 0.001^*^
*Laboratory values*							
Creatinine, μmol/l	83.0 [70.0–97.0]	48	1	91.0 [75.0–114]	29	0	< 0.001^*^
*Echocardiography*							
LVEF	55.0 [55.0–56.0]	72	1	55.0 [41.0–55.0]	204	1	< 0.001^*^
PASP, mm Hg	25.0 [25.0–25.0]	627	8	25.0 [25.0–31.0]	2040	14	< 0.001^*^

Data are presented as *n* (%) or median [interquartile range]

*P value of < 0.05 was considered statistically significant

*BMI* body mass index, *CCS* Canadian Cardiovascular Society classification, *EuroSCORE* European System for Cardiac Operative Risk Evaluation, *LVEF* Left Ventricular Ejection Fraction, *MI* Myocardial Infarction, *NYHA* New York Heart Association functional classification, *PASP* pulmonary arterial systolic pressure, *SAVR* surgical aortic valve replacement, *TAVI* transcatheter aortic valve implantation

TAVI patients showed higher prevalences of COPD, diabetes, AF, dialysis, previous stroke, unstable angina and recent MI than SAVR patients. In addition, they had more frequently undergone previous cardiac surgery, had higher creatinine levels, lower LVEF and higher PASP. After stratifying patients by age, these higher rates in TAVI remained statistically significant, except for diabetes and dialysis in the older age group. SAVR patients, on the other hand, had higher rates of endocarditis, a critical pre-operative condition and higher urgency, which were consistent through all age groups. Tables S2 and S3 in the Electronic Supplementary Material show baseline characteristics of both TAVI and SAVR cohorts in early (2013–2016) and recent (2017–2021) years. Several changes in baseline characteristics were observed over time, which were largely similar between TAVI and SAVR cohorts. Regarding trends in older age groups, the proportions of female sex, age and EuroSCORE II increased in older age groups in both cohorts. The prevalences of COPD, AF, dialysis, recent MI and previous cardiac surgery declined in older age groups in both cohorts and thus were highest in younger age groups (see Table S4 in Electronic Supplementary Material).

In SAVR patients, the prevalence of NYHA class III/IV increased with higher age (*p* for trend < 0.001), while it did not change and remained high in all age groups for TAVI patients. Rates of poor mobility, diabetes, previous stroke, recent MI, critical pre-operative condition and urgency decreased in older age groups for TAVI patients, while this was not the case for SAVR patients. LVEF increased across age groups in TAVI patients but not in SAVR patients PASP increased in subsequent age groups for both cohorts.

### Mortality and aortic valve re-intervention at 5-year follow-up

**Fig. 2:**
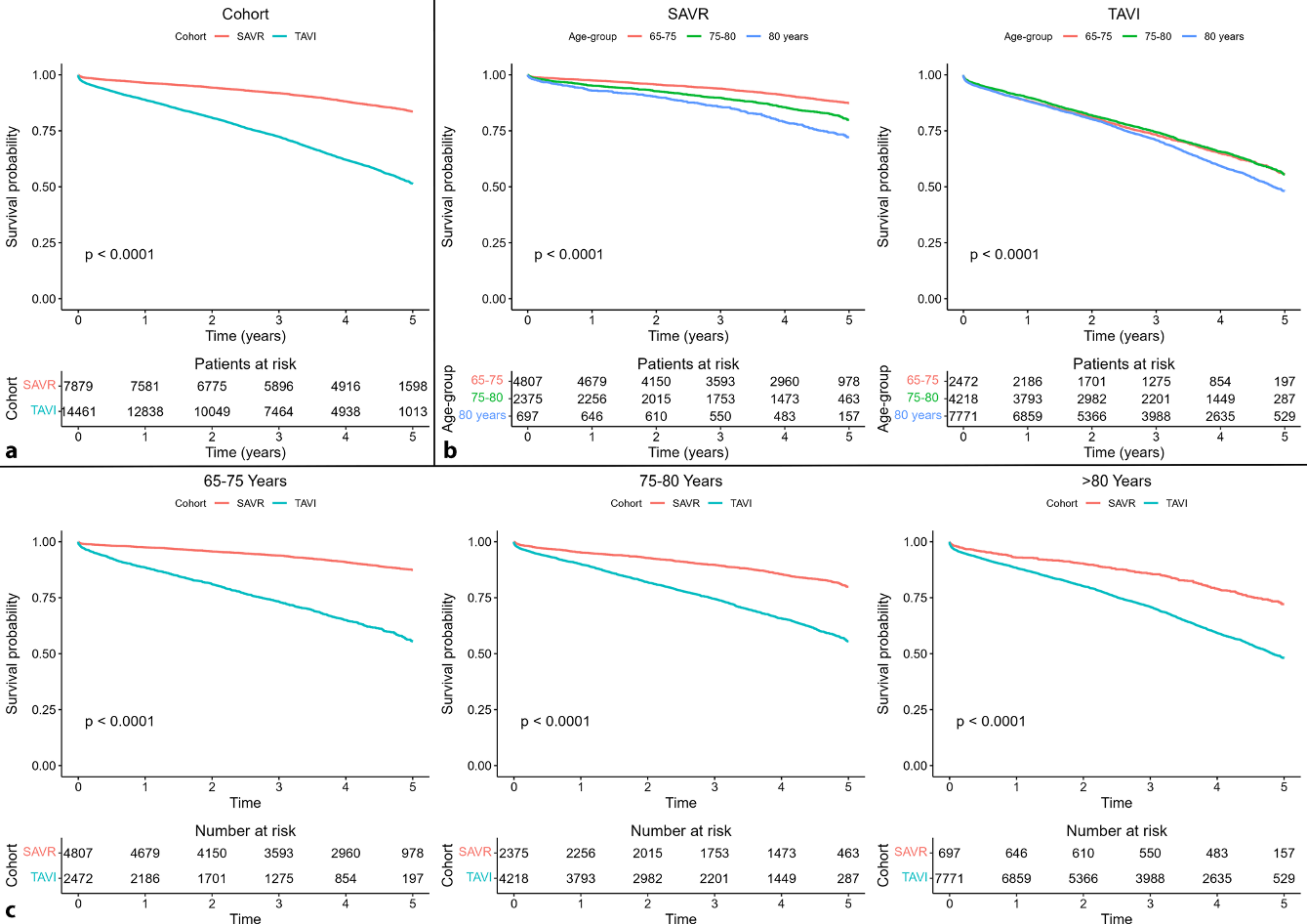
Kaplan–Meier survival curves of 5‑year mortality after surgical aortic valve replacement (*SAVR*) versus transcatheter aortic valve implantation (*TAVI*). **a** Total cohort, **b** SAVR-treated patients only, stratified by age group, **c** TAVI-treated patients only, stratified by age group, **d** youngest age group (65–75 years), stratified by cohort, **e** middle age group (75–80 years), stratified by cohort, and **f** oldest age group (> 80 years), stratified by cohort

After a median follow-up time of 1617 days, 1012/7879 (12.8%) SAVR patients died. In contrast, after 1121 days of follow up, 5140/14,461 (35.5%) TAVI patients died (Tab. 2). Median survival was shorter after TAVI (685 days; IQR: 250–1160 vs 874 days; IQR: 317–1356; *p* < 0.001 (Fig. 2)). This trend was also seen in both early and recent years (see Tables S2 and S3 in Electronic Supplementary Material) and when including only transfemoral TAVI patients versus SAVR patients (see Table S5 in Electronic Supplementary Material). However, there was no interaction between time, cohort and outcomes (see Table S6 in Electronic Supplementary Material). Aortic valve re-intervention rates were low (135 (1.9%) in SAVR patients vs 114 (0.9%) in TAVI patients; *p* < 0.001), and the time to aortic valve re-intervention was shorter in TAVI compared with SAVR (52 vs 338 days; *p* < 0.001).

**Table 2:** Outcomes

Variable	SAVR (*N* = 7879)	TAVI (*N* = 14,461)	*P*-value
*Mortality n (%)*	1012 (12.8)	5140 (35.5)	< 0.001^*^
Per 100 patient-years	3.35 (3.14–3.56)	11.99 (11.67–12.33)	–
Time to mortality (median [IQR])	873.5 [316.8–1356.0]	685.0 [249.8–1160.0]	< 0.001^*^
1‑year mortality *n *(%)	283 (3.6)	1615 (11.2)	< 0.001^*^
*Aortic valve re-intervention n (%)*	133 (1.9)	112 (0.9)	< 0.001^*^
Time to re-intervention (median [IQR])	336.0 [110.0–804.0]	57.5 [11.8–252.0]	< 0.001^*^
1‑year re-intervention *n *(%)	70 (1.0)	88 (0.7)	0.014^*^
Days of follow up (median [IQR])	1617.0 [1088.0–1792.0]	1121.0 [627.0–1602.0]	< 0.001^*^

*P value of < 0.05 was considered statistically significant

Time-to-event by log-rank test

Comparing TAVI and SAVR patients who died during follow-up, showed that TAVI patients were older and more frequently female and had lower BMI, more often NYHA class III/IV at the time of the procedure, poor mobility and higher EuroSCORE II (see Table S7 in Electronic Supplementary Material). TAVI patients who had died had a higher prevalence of chronic lung disease, AF, stroke and previous cardiac surgery. LVEF was lower in TAVI patients, whereas PASP was higher. SAVR patients, in contrast, more frequently were in critical pre-operative condition and had previous thoracic aortic surgery and higher urgency.

### Outcomes stratified by age

Mortality rates increased with increasing age for both procedures, but this was more pronounced after SAVR than TAVI (9.7% for 65–75 years, 15.7% for 75–80 years and 24.5% for > 80 years in SAVR; *p* < 0.001, compared with 32.8%, 32.1% and 38.3%, respectively, in TAVI; *p* < 0.001) (Tab. 3, and see Tables S8 and S9 in Electronic Supplementary Material). Interaction analysis between cohort and age group revealed that the hazard associated with TAVI decreased relative to SAVR in higher age groups (see Table S10 in Electronic Supplementary Material). Aortic valve re-intervention rates were low and declined with increasing age (2.4%, 1.4% and 0.5% in SAVR; *p* < 0.001, compared with 1.5%, 0.9% and 0.7% in TAVI; *p* < 0.001).

**Table 3:** Outcomes per age group

			Age group			
Outcome	Cohort	Total	65–75 years	75–80 years	> 80 years	*P*-value
Mortality	SAVR	1012 (12.8)	467 (9.7)	374 (15.7)	171 (24.5)	< 0.001^*^
	TAVI	5140 (35.5)	811 (32.8)	1354 (32.1)	2975 (38.3)	< 0.001^*^
Aortic valve re-intervention	SAVR	133 (1.9)	101 (2.4)	29 (1.4)	3 (0.5)	0.001^*^
	TAVI	112 (0.9)	32 (1.5)	34 (0.9)	46 (0.7)	0.001^*^

Crude survival and aortic valve re-intervention data, maximally 5 years after procedure

Data are presented as *n* (%)

^*^ *P* value of < 0.05 was considered statistically significant

## Discussion

The main findings of this large retrospective analysis of a prospective, nationwide, real-world cohort of patients undergoing SAVR or TAVI were: (1) SAVR and TAVI patients in the Netherlands differed substantially in baseline characteristics, as TAVI patients had more comorbidities, most notably in the lowest age group (65–75 years); (2) mortality rates were higher after TAVI compared with SAVR; (3) mortality rates after SAVR increased with age, but remained high in the TAVI population across all age groups, indicating that younger patients undergoing TAVI had more comorbidities; and (4) aortic re-intervention rates were low after both procedures.

### Differences in clinical profiles

The differences in age, sex and prevalence of comorbidities, such as COPD, diabetes, AF and previous stroke, we observed are in line with results from other nationwide registries in the literature [7–9]. The significantly higher age of TAVI patients was expected and can be explained by the use of local, national and international guidelines. In the Netherlands, TAVI procedures are only reimbursed for patients > 80 years or those at high surgical risk [5]. Our study showed that several characteristics of both patients undergoing SAVR or TAVI changed over time. In addition, there was no change in baseline characteristics before and after the Dutch indication document for reimbursement was effectuated in 2021, as has been shown in a recent publication based on the same NHR registries [12]. The same study indicated that age > 80 years was the most common reason for choosing TAVI in the Netherlands. However, even within the stratified age groups, there were still several remarkable differences between TAVI and SAVR patients. For example, the prevalences of all comorbidities within each age category were greater in the TAVI group. This difference between TAVI and SAVR was most prominent in the younger age group, indicating that younger patients with more comorbidities or a high-risk profile underwent TAVI. This is perhaps not surprising given that patients with more comorbidities tend to be declined for surgical treatment by the Heart Team. Currently, there is an emerging trend towards treating younger patients with fewer comorbidities with TAVI in many European countries and in the USA [13]. Several low-risk trials involving younger and relatively healthier patients are currently in the follow-up phase and should give more insight into the outcomes in this patient group in the coming years [1, 3, 4, 14–16].

### Long-term mortality rates

In patients undergoing SAVR, long-term mortality rates clearly increased with age, which is in line with previously published data [17]. This trend was less obvious in patients undergoing TAVI. This may be explained again by the common practice that patients with a high rate of comorbidities are more likely to undergo TAVI and these patients have lower life expectancy, even when the procedure is performed at younger age. The cause of death was unknown in the current registry. However, it has been shown that long-term mortality after both procedures is generally mostly non-valve-related [18–20].

### Long-term aortic re-intervention rates

The aortic valve re-intervention rate was low across all age groups and slightly lower compared with other (randomised) cohorts [1, 21]. The aortic valve re-intervention rate was lowest in older patients, possibly due to competing risks; either older patients die before the valve is able to degenerate or they are too frail to undergo a second AVR procedure. Another possibility is the earlier and rapid degeneration of bioprosthetic valves in younger patients, although the evidence is conflicting [22, 23]. In this registry, we did not have information on the reasons for withholding aortic valve re-intervention, and other cohorts of patients undergoing TAVI or SAVR unfortunately also did not capture this information [24, 25]. Information on valvular degeneration and symptomatology is essential in the follow-up of the predominantly older AVR population, as symptomatology may frequently be determined by comorbidities. When post-AVR patients become symptomatic, the decision for aortic valve re-intervention requires a balanced consideration of expected decrease of symptoms and expected risk of procedural complications.

### Study limitations

This study has some limitations. First, there was a high risk of residual confounding due to the selective collection of variables. Second, due to the eligibility criteria for TAVI in the Netherlands, there was a selection bias, as younger patients without risk factors are declined for TAVI. The aforementioned low-risk trials should provide new information regarding the outcomes of younger patients. Lastly, by design, we excluded patients with additional procedures and SAVR patients with concomitant coronary artery disease, inducing a potential additional bias.

### Implications

There are substantial differences in baseline characteristics between patients undergoing SAVR and TAVI. Based on our study and other previously reported data [7, 25], younger patients with fewer comorbidities seem to be underrepresented in Dutch and other TAVI cohorts. It could be questioned whether comparison of outcomes after both procedures is even possible. Real-world and randomised trials of patients aged < 75 years and with fewer comorbidities are needed and should include long-term follow-up. In addition, mortality may not be an appropriate long-term outcome comparator since this endpoint can be driven by non-valvular-related factors and other outcomes, such as decrease of symptoms, hospitalisation for heart failure, valve degeneration and re-intervention, may be more meaningful.

## Conclusion

Patients who underwent SAVR and TAVI in the Netherlands differed substantially with respect to their baseline characteristics, even when stratified by age. Mortality rates were highest in TAVI patients, but this difference declined with increasing age, whereas aortic re-intervention was more frequently seen in SAVR patients.

## Supplementary Information

Table S1 Baseline characteristics, stratified by age-group and cohort

Table S2 Differences in demographics and outcomes per time, separately for each cohort

Table S3 Difference in demographics and outcomes between cohorts, for each time period

Table S4 Baseline per age-group, stratified by cohort

Table S5 Demographics and outcomes per time period, for only transfemoral TAVI

Table S6 Baseline per age-group, stratified by cohort

Table S7 Baseline by outcomes, stratified by cohort

Table S8 Outcomes per age-group, stratified by cohort

Table S9 Mortality per 100 patient years

Table S10 Hazard ratios of cohort and age-group
