# Supplementary material for: Long-term age-stratified outcomes after surgical and transcatheter aortic valve replacement: a Dutch cohort study
Source: Neth Heart J. 2025 Apr 11;33(5):172–9. doi: 10.1007/s12471-025-01944-5 (PMC12014882; doi:10.1007/s12471-025-01944-5)
Supplement: Supplementary file 1 — Table S1 Baseline characteristics, stratified by age-group and cohort [file 12471_2025_1944_MOESM1_ESM.docx]

**Table S1** Baseline characteristics, stratified by age-group and cohort

| Age-group | 65-75 years | | | 75-80 years | | | | >80 years | | |  |
| --- | --- | --- | --- | --- | --- | --- | --- | --- | --- | --- | --- |
| Cohort | SAVR | TAVI |  | SAVR | TAVI |  | SAVR | | TAVI |  |  |
| N | 4870 | 2472 |  | 2375 | 4218 |  | 697 | | 7771 |  |  |
| Demographics | | | | | | | | | | | |
| Age*, years* | 70.0 [67.0-72.0] | 72.0 [69.0-73.0] | <0.001* | 77.0 [76.0-79.0] | 78.0 [76.0-79.0] | <0.001* | 82.0 [81.0-83.0] | | 84.0 [82.0-86.0] | <0.001* |  |
| Sex *(Female)* | 1945 (40.5)) | 1034 (41.8) | 0.272 | 1108 (46.7) | 1944 (46.1) | 0.678 | 394 (56) | | 4254 (54.7) | 0.385 |  |
| BMI | 27.6 [24.8-30.9] | 28.0 [24.5-32.7] | <0.001* | 27.0 [24.6-29.7] | 27.1 [24.3-30.5] | 0.213 | 26.6 [24.2-29.4] | | 25.8 [23.6-28.7] | <0.001* |  |
| NYHA Class III/IV | 1122 (30.2) | 1321 (57.6) | <0.001* | 598 (33.4) | 2212 (56.9) | <0.001* | 174 (37.6) | | 4179 (57.9) | <0.001* |  |
| CCS Class IV | 43 (1.04) | 54 (2.49) | <0.001* | 9 (0.44) | 76 (2.07) | <0.001* | 9 (1.65) | | 172 (2.52) | 0.265 |  |
| Poor Mobility | 123 (3.02) | 267 (12.9) | <0.001* | 70 (3.54) | 389 (11.2) | <0.001* | 23 (4.45) | | 525 (8.21) | 0.003* |  |
| EuroSCORE II | 1.24 [0.97-1.74] | 2.56 [1.53-4.60] | <0.001* | 1.70 [1.35-2.48] | 2.77 [1.79-4.93] | <0.001* | 2.38 [1.75-3.60] | | 3.71 [2.38-5.85] | <0.001* |  |
| Comorbidities | | | | | | | | | | | |
| Chronic Lung Disease | 637 (13.3) | 685 (27.7) | <0.001* | 294 (12.4) | 922 (21.9) | <0.001* | 64 (9.18) | | 1211 (15.6) | <0.001* |  |
| Diabetes | 1016 (21.4) | 908 (37.1) | <0.001* | 515 (22.0) | 1288 (30.9) | <0.001* | 139 (20.3) | | 1693 (22.1) | 0.297 |  |
| Atrial Fibrillation | 283 (8.93) | 86 (24.0) | <0.001* | 220 (14.5) | 179 (31.6) | <0.001* | 62 (16.9) | | 343 (31.6) | <0.001* |  |
| Dialysis | 19 (0.45) | 51 (2.09) | <0.001* | 3 (0.15) | 46 (1.10) | <0.001* | 0 (0.00) | | 32 (0.42) | 0.267 |  |
| Stroke | 228 (5.08) | 308 (12.5) | <0.001* | 104 (4.70) | 446 (10.6) | <0.001* | 28 (4.30) | | 756 (9.74) | <0.001* |  |
| Cardiac status | | | | | | | | | | | |
| Unstable Angina | 9 (0.19) | 11 (0.45) | 0.075 | 1 (0.04) | 11 (0.26) | 0.067 | 1 (0.14) | | 22 (0.29) | 1.000 |  |
| Recent MI | 60 (1.25) | 59 (2.40) | <0.001* | 23 (0.97) | 82 (1.96) | 0.003* | 4 (0.57) | | 134 (1.74) | 0.031* |  |
| Previous Cardiac Surg*.* | 219 (4.56) | 574 (23.6) | <0.001* | 62 (2.61) | 760 (18.3) | <0.001* | 21 (3.01) | | 881 (11.5) | <0.001* |  |
| Thoracic Aortic Surg*.* | 2 (0.04) | 1 (0.04) | 1.000 | 1 (0.04) | 0 (0.00) | 0.365 | 0 (0.00) | | 4 (0.05) | 1.000 |  |
| Endocarditis | 159 (3.31) | 0 (0.00) | <0.001* | 41 (1.73) | 1 (0.02) | <0.001* | 12 (1.72) | | 1 (0.01) | <0.001* |  |
| Critical Pre-op. Cond. | 48 (1.00) | 26 (1.06) | 0.908 | 17 (0.72) | 12 (0.29) | 0.020* | 5 (0.72) | | 19 (0.25) | 0.044* |  |
| Urgency | 600 (13.0) | 277 (11.4) | 0.053 | 253 (11.2) | 357 (8.58) | 0.001* | 91 (14.2) | | 660 (8.63) | <0.001* |  |
| Laboratory values | | | | | | | | | | | |
| Creatinine *(μmol/l)* | 82.0 [70.0-96.0] | 90.0 [73.0-115] | <0.001* | 83.0 [70.0-98.0] | 91.0 [75.0-113] | <0.001* | 84.0 [71.0-100] | | 92.0 [75.0-114] | <0.001* |  |
| Echocardiography | | | | | | | | | | | |
| LVEF *(%)* | 55.0 [55.0-56.0] | 55.0 [40.0-55.0] | <0.001* | 55.0 [55.0-56.0] | 55.0 [40.0-55.0] | <0.001* | 55.0 [55.0-56.0] | | 55.0 [45.0-55.0] | <0.001* |  |
| PASP *(mmHg)* | 25.0 [25.0-25.0] | 25.0 [25.0-30.0] | <0.001* | 25.0 [25.0-25.0] | 25.0 [25.0-29.0] | <0.001* | 25.0 [25.0-25.0] | | 25.0 [25.0-33.0] | <0.001* |  |
| Data are presented as n (%) or median [interquartile range].  * P value of <0.05 is considered statistically significant  BMI: Body Mass Index; CCS: Canadian Cardiovascular Society Classification; EuroSCORE: European System for Cardiac Operative Risk Evaluation; LVEF: Left Ventricular Ejection Fraction; MI: Myocardial Infarction; NYHA: New York Heart Association Functional Classification; PASP: Pulmonary Artery Pressure; SAVR: Surgical Aortic Valve Replacement; TAVI: Transcatheter Aortic Valve Implantation | | | | | | | | | | | |
