# Supplementary material for: Long-term age-stratified outcomes after surgical and transcatheter aortic valve replacement: a Dutch cohort study
Source: Neth Heart J. 2025 Apr 11;33(5):172–9. doi: 10.1007/s12471-025-01944-5 (PMC12014882; doi:10.1007/s12471-025-01944-5)
Supplement: Supplementary file 2 — Table S2 Differences in demographics and outcomes per time, separately for each cohort [file 12471_2025_1944_MOESM2_ESM.docx]

**Table S2** Differences in demographics and outcomes per time, separately for each cohort

| Cohort | SAVR | | | TAVI | | |  |
| --- | --- | --- | --- | --- | --- | --- | --- |
| Years | **2013-2016** | **2017-2021** | **P** | **2013-2016** | **2017-2021** | p |  |
| N | N=4029 | N=3850 |  | N=4222 | N=10239 |  |  |
| Demographics | | | | | | | |
| Age*, years* | 73.0 [69.0-78.0] | 72.0 [69.0-76.0] | **<0.001*** | 81.0 [77.0-85.0] | 81.0 [76.0-84.0] | **<0.001*** |  |
| Sex *(Female)* | 1819 (45.1) | 1628 (43.3) | **0.011*** | 2238 (53.0) | 4994 (48.8) | **<0.001*** |  |
| BMI | 27.4 [24.8-30.4] | 27.2 [24.6-30.5] | 0.190 | 26.5 [24.0-30.0] | 26.4 [23.9-29.7] | 0.219 |  |
| NYHA Class III/IV | 876 (36.5) | 1018 (28.5) | **<0.001*** | 2456 (66.3) | 5256 (54.2) | **<0.001*** |  |
| CCS Class IV | 32 (1.08) | 29 (0.77) | 0.218 | 67 (2.35) | 235 (2.39) | 0.949 |  |
| Poor Mobility | 160 (5.74) | 56 (1.48) | **<0.001*** | 211 (9.39) | 970 (10.0) | 0.395 |  |
| EuroSCORE II | 1.55 [1.13-2.34] | 1.38 [1.05-1.97] | **<0.001*** | 4.00 [2.36-6.54] | 3.11 [1.97-5.14] | **<0.001*** |  |
| Comorbidities | | | | | | | |
| Chronic Lung Disease | 554 (13.8) | 441 (11.5) | **0.002*** | 971 (23.1) | 1847 (18.1) | **<0.001*** |  |
| Diabetes | 919 (23.2) | 751 (19.6) | **<0.001*** | 1147 (28.2) | 2742 (26.8) | 0.103 |  |
| Atrial Fibrillation | 213 (12.1) | 352 (10.7) | 0.162 | 0 (.) | 608 (30.2) | . |  |
| Dialysis | 14 (0.47) | 8 (0.21) | 0.109 | 61 (1.51) | 68 (0.67) | **<0.001*** |  |
| Stroke | 159 (4.42) | 201 (5.36) | 0.068 | 507 (12.0) | 1003 (9.80) | **<0.001*** |  |
| Cardiac Status | | | | | | | |
| Unstable Angina | 4 (0.10) | 7 (0.18) | 0.501 | 4 (0.10) | 40 (0.39) | **0.008*** |  |
| Recent MI | 39 (0.97) | 48 (1.25) | 0.287 | 67 (1.62) | 208 (2.04) | 0.114 |  |
| Previous Cardiac Surg*.* | 204 (5.06) | 98 (2.55) | **<0.001*** | 786 (19.5) | 1429 (14.0) | **<0.001*** |  |
| Thoracic Aortic Surg*.* | 2 (0.05) | 1 (0.03) | 1.000 | 3 (0.08) | 2 (0.02) | 0.135 |  |
| Endocarditis | 89 (2.21) | 123 (3.20) | **0.009*** | 1 (0.03) | 1 (0.01) | 0.455 |  |
| Critical Pre-op. Cond. | 33 (0.82) | 37 (0.96) | 0.586 | 21 (0.51) | 36 (0.35) | 0.234 |  |
| Urgency | 367 (9.98) | 577 (15.0) | **<0.001*** | 353 (8.75) | 941 (9.22) | 0.398 |  |
| Laboratory values | | | | | | | |
| Creatinine *(μmol/l)* | 83.0 [70.0-98.0] | 82.0 [70.0-96.0] | 0.175 | 93.0 [76.0-117] | 91.0 [74.0-113] | **<0.001*** |  |
| Echocardiography | | | | | | | |
| LVEF *%* | 55.0 [55.0-60.0] | 55.0 [55.0-56.0] | 0.400 | 55.0 [40.0-55.0] | 55.0 [43.0-55.0] | **0.004*** |  |
| PASP *(mmHg)* | 25.0 [25.0-25.0] | 25.0 [25.0-25.0] | **<0.001*** | 25.0 [25.0-34.0] | 25.0 [25.0-30.0] | **<0.001*** |  |
| Outcomes (crude) | | | | | | | |
| 1-year mortality | 149 (3.70) | 134 (3.48) | 0.647 | 573 (13.6) | 1042 (10.2) | **<0.001*** |  |
| 5-year mortality | 624 (15.5) | 388 (10.1) | **<0.001*** | 2047 (48.5) | 3093 (30.2) | **<0.001*** |  |
| 1-year re-intervention | 33 (1.05) | 37 (1.00) | 0.937 | 25 (0.72) | 63 (0.67) | 0.852 |  |
| 5-year re-intervention | 65 (2.06) | 68 (1.83) | 0.552 | 38 (1.09) | 74 (0.78) | 0.118 |  |
| Data are presented as n (%) or median [interquartile range].  * P value of <0.05 is considered statistically significant.  BMI: Body Mass Index; CCS: Canadian Cardiovascular Society Classification; EuroSCORE: European System for Cardiac Operative Risk Evaluation; LVEF: Left Ventricular Ejection Fraction; MI: Myocardial Infarction; NYHA: New York Heart Association Functional Classification; PASP: Pulmonary Arterial systolicPressure; SAVR: Surgical Aortic Valve Replacement; TAVI: Transcatheter Aortic Valve Implantation | | | | | | | |
