# Supplementary material for: Long-term age-stratified outcomes after surgical and transcatheter aortic valve replacement: a Dutch cohort study
Source: Neth Heart J. 2025 Apr 11;33(5):172–9. doi: 10.1007/s12471-025-01944-5 (PMC12014882; doi:10.1007/s12471-025-01944-5)
Supplement: Supplementary file 4 — Table S4 Baseline per age-group, stratified by cohort [file 12471_2025_1944_MOESM4_ESM.docx]

**Table S4** Baseline per age-group, stratified by cohort

| Cohort | SAVR | | | | TAVI | | | |  |
| --- | --- | --- | --- | --- | --- | --- | --- | --- | --- |
| Age-group | **65-75 years** | **75-80 years** | **>80 years** | P for  trend^†^ | **65-75 years** | **75-80 years** | **>80 years** | P for  trend^†^ | **Trend in cohort** |
| n | 4807 | 2375 | 697 |  | 2472 | 4218 | 7771 |  |  |
| Demographics | | | | | | | | | |
| Age*, years* | 70.0 [67.0-72.0] | 77.0 [76.0-79.0] | 82.0 [81.0-83.0] | **0.001*** | 72.0 [69.0-73.0] | 78.0 [76.0-79.0] | 84.0 [82.0-86.0] | **0.001*** | **Both** |
| Sex *(Female)* | 1945 (40.5) | 1108 (46.7) | 394 (56.5) | **0.001*** | 1034 (41.8) | 1944 (46.1) | 4254 (54.7) | **0.001*** | **Both** |
| BMI, | 27.6 [24.8-30.9] | 27.0 [24.6-29.7] | 26.6 [24.2-29.4] | **<0.001*** | 28.0 [24.5-32.7] | 27.1 [24.3-30.5] | 25.8 [23.6-28.7] | **<0.001*** | **Both** |
| NYHA Class III/IV | 1122 (30.2) | 598 (33.4) | 174 (37.6) | **<0.001*** | 1321 (57.6) | 2212 (56.9) | 4179 (57.9) | 0.570 | **SAVR** |
| CCS Class IV | 43 (1.04) | 9 (0.44) | 9 (1.65) | 0.791 | 54 (2.49) | 76 (2.07) | 172 (2.52) | 0.593 | None |
| Poor Mobility | 123 (3.02) | 70 (3.54) | 23 (4.45) | 0.066 | 267 (12.9) | 389 (11.2) | 525 (8.21) | **<0.001*** | **TAVI** |
| EuroSCORE II | 1.24 [0.97-1.74] | 1.70 [1.35-2.48] | 2.38 [1.75-3.60] | **<0.001*** | 2.56 [1.53-4.60] | 2.77 [1.79-4.93] | 3.71 [2.38-5.85] | **<0.001*** | **Both** |
| Comorbidities | | | | | | | | | |
| Chronic Lung Disease | 637 (13.3) | 294 (12.4) | 64 (9.18) | **0.005*** | 685 (27.7) | 922 (21.9) | 1211 (15.6) | **0.001*** | **Both** |
| Diabetes | 1016 (21.4) | 515 (22.0) | 139 (20.3) | 0.9875 | 908 (37.1) | 1288 (30.9) | 1693 (22.1) | **0.001*** | **TAVI** |
| Atrial Fibrillation | 283 (8.93) | 220 (14.5) | 62 (16.9) | **<0.001*** | 86 (24.0) | 179 (31.6) | 343 (31.6) | **0.018** | **Both** |
| Dialysis | 19 (0.45) | 3 (0.15) | 0 (0.00) | **0.018*** | 51 (2.09) | 46 (1.10) | 32 (0.42) | **<0.001*** | **Both** |
| Stroke | 228 (5.08) | 104 (4.70) | 28 (4.30) | 0.317 | 308 (12.5) | 446 (10.6) | 756 (9.74) | **<0.001*** | **TAVI** |
| Cardiac Status | | | | | | | | | |
| Unstable Angina | 9 (0.19) | 1 (0.04) | 1 (0.14) | 0.296 | 11 (0.45) | 11 (0.26) | 22 (0.29) | 0.305 | None |
| Recent MI | 60 (1.25) | 23 (0.97) | 4 (0.57) | 0.079 | 59 (2.40) | 82 (1.96) | 134 (1.74) | **0.038*** | **TAVI** |
| Previous Cardiac Surg*.* | 219 (4.56) | 62 (2.61) | 21 (3.01) | **<0.001*** | 574 (23.6) | 760 (18.3) | 881 (11.5) | **<0.001*** | **Both** |
| Thoracic Aortic Surg*.* | 2 (0.04) | 1 (0.04) | 0 (0.00) | 0.700 | 1 (0.04) | 0 (0.00) | 4 (0.05) | 0.493 | None |
| Endocarditis | 159 (3.31) | 41 (1.73) | 12 (1.72) | **<0.001*** | 0 (0.00) | 1 (0.02) | 1 (0.01) | 0.802 | **SAVR** |
| Critical Pre-op. Cond. | 48 (1.00) | 17 (0.72) | 5 (0.72) | 0.232 | 26 (1.06) | 12 (0.29) | 19 (0.25) | **<0.001*** | **TAVI** |
| Urgency | 600 (13.0) | 253 (11.2) | 91 (14.2) | 0.602 | 277 (11.4) | 357 (8.58) | 660 (8.63) | **<0.001*** | **TAVI** |
| Laboratory values | | | | | | | | | |
| Creatinine *(μmol/l)* | 82.0 [70.0-96.0] | 83.0 [70.0-98.0] | 84.0 [71.0-100] | **0.008*** | 90.0 [73.0-115] | 91.0 [75.0-113] | 92.0 [75.0-114] | 0.211 | **SAVR** |
| Echocardiography | | | | | | | | | |
| LVEF *(%)* | 55.0 [55.0-56.0] | 55.0 [55.0-56.0] | 55.0 [55.0-56.0] | 0.953 | 55.0 [40.0-55.0] | 55.0 [40.0-55.0] | 55.0 [45.0-55.0] | **<0.001*** | **TAVI** |
| PASP (*mmHg)* | 25.0 [25.0-25.0] | 25.0 [25.0-25.0] | 25.0 [25.0-25.0] | **<0.001*** | 25.0 [25.0-30.0] | 25.0 [25.0-29.0] | 25.0 [25.0-33.0] | **0.015*** | **Both** |
| Data are presented as n (%) or median [interquartile range]. ^†^ Pearson test and Spearman test for normal and non-normal distributed continuous variables, and Mantel-Haenszel test for categorical variables.* P value of <0.05 is considered statistically significant  BMI: Body Mass Index; CCS: Canadian Cardiovascular Society Classification; EuroSCORE: European System for Cardiac Operative Risk Evaluation; LVEF: Left Ventricular Ejection Fraction; MI: Myocardial Infarction; NYHA: New York Heart Association Functional Classification; PASP: Pulmonary Arterial systolicy Pressure; SAVR: Surgical Aortic Valve Replacement; TAVI: Transcatheter Aortic Valve Implantation | | | | | | | | | |
