# Supplementary material for: Long-term age-stratified outcomes after surgical and transcatheter aortic valve replacement: a Dutch cohort study
Source: Neth Heart J. 2025 Apr 11;33(5):172–9. doi: 10.1007/s12471-025-01944-5 (PMC12014882; doi:10.1007/s12471-025-01944-5)
Supplement: Supplementary file 5 — Table S5 Demographics and outcomes per time period, for only transfemoral TAVI [file 12471_2025_1944_MOESM5_ESM.docx]

**Table S5** Demographics and outcomes per time period, for only transfemoral TAVI

|  | | | |
| --- | --- | --- | --- |
| Years | **SAVR** | **TAVI-TF** | **p** |
| N | **N=7879** | **N=11882** |  |
| Demographics | | | |
| Age*, years* | 73.0 [69.0-77.0] | 81.0 [77.0-85.0] | **<0.001*** |
| Sex *(Female)* | 3447 (43.7%) | 6049 (50.9) | **<0.001*** |
| BMI | 27.3 [24.7-30.4] | 26.6 [24.0-29.9] | **<0.001*** |
| NYHA Class III/IV | 1894 (31.7) | 6308 (57.0) | **<0.001*** |
| CCS Class IV | 61 (0.91) | 229 (2.19) | **<0.001*** |
| Poor Mobility | 216 (3.29) | 929 (9.42) | **<0.001*** |
| EuroSCORE II | 1.44 [1.08-2.13] | 3.12 [1.99-5.13] | **<0.001*** |
| Comorbidities | | | |
| Chronic Lung Disease | 995 (12.6) | 2087 (17.6) | **<0.001*** |
| Diabetes | 1670 (21.4) | 3122 (26.6) | **<0.001*** |
| Atrial Fibrillation | 565 (11.2) | 546 (30.0) | **<0.001*** |
| Dialysis | 22 (0.33) | 102 (0.87) | **<0.001*** |
| Stroke | 360 (4.90) | 1194 (10.1) | **<0.001*** |
| Cardiac Status | | | |
| Unstable Angina | 11 (0.14) | 32 (0.27) | 0.072 |
| Recent MI | 87 (1.11) | 208 (1.76) | **<0.001*** |
| Previous Cardiac Surg*.* | 302 (3.83) | 1719 (14.7) | **<0.001*** |
| Thoracic Aortic Surg*.* | 3 (0.04) | 4 (0.03) | 1.000 |
| Endocarditis | 212 (2.69) | 2 (0.02) | **<0.001*** |
| Critical Pre-op. Cond. | 70 (0.89) | 40 (0.34) | **<0.001*** |
| Urgency | 944 (12.5) | 1053 (8.99) | **<0.001*** |
| Laboratory values | | | |
| Creatinine *(μmol/l)* | 83.0 [70.0-97.0] | 90.0 [74.0-113] | **<0.001*** |
| Echocardiography | | | |
| LVEF *%* | 55.0 [55.0-56.0] | 55.0 [42.0-55.0] | **<0.001*** |
| PASP *(mmHg)* | 25.0 [25.0-25.0] | 25.0 [25.0-32.0] | **<0.001*** |
| Outcomes (crude) | | | |
| 1-year mortality | 283 (3.59) | 1177 (9.91) | **<0.001*** |
| 5-year mortality | 1012 (12.8) | 3940 (33.2) | **<0.001*** |
| 1-year re-intervention | 70 (1.02) | 76 (0.72) | **0.042*** |
| 5-year re-intervention | 133 (1.94) | 98 (0.93) | **<0.001*** |
| Data are presented as n (%) or median [interquartile range].  * P value of <0.05 is considered statistically significant.  BMI: Body Mass Index; CCS: Canadian Cardiovascular Society Classification; EuroSCORE: European System for Cardiac Operative Risk Evaluation; LVEF: Left Ventricular Ejection Fraction; MI: Myocardial Infarction; NYHA: New York Heart Association Functional Classification; PASP: pulmonary arterial systolic pressure; SAVR: Surgical Aortic Valve Replacement; TAVR: Transcatheter Aortic Valve Implantation, TF: Transfemoral | | | |
