# Supplementary material for: Long-term age-stratified outcomes after surgical and transcatheter aortic valve replacement: a Dutch cohort study
Source: Neth Heart J. 2025 Apr 11;33(5):172–9. doi: 10.1007/s12471-025-01944-5 (PMC12014882; doi:10.1007/s12471-025-01944-5)
Supplement: Supplementary file 6 — Table S6 Baseline per age-group, stratified by cohort [file 12471_2025_1944_MOESM6_ESM.docx]

**Table S6** Baseline per age-group, stratified by cohort

|  | **HR (95% CI)** | **p-value** | **p-interaction^†^** |
| --- | --- | --- | --- |
| **TAVI**(versus SAVR, 2013-2016) | 3.96 (3.62-4.33) | **<0.001** | 0.272 |
| **TAVI**(versus SAVR, 2017-2021) | 3.66 (3.30-4.01) | **<0.001** |  |
| **^†^Interaction between cohort and year.**  **Analysis shows stratified years because of non-proportional hazards for age-group (by Schoenfeld Residuals)** | | | |
