# Supplementary material for: Long-term age-stratified outcomes after surgical and transcatheter aortic valve replacement: a Dutch cohort study
Source: Neth Heart J. 2025 Apr 11;33(5):172–9. doi: 10.1007/s12471-025-01944-5 (PMC12014882; doi:10.1007/s12471-025-01944-5)
Supplement: Supplementary file 7 — Table S7 Baseline by outcomes, stratified by cohort [file 12471_2025_1944_MOESM7_ESM.docx]

**Table S7** Baseline by outcomes, stratified by cohort

| 5-year mortality | No | | | Yes | | |
| --- | --- | --- | --- | --- | --- | --- |
| Cohort | SAVR | TAVI | p | SAVR | TAVI | p |
| N | 6867 | 9321 |  | 1012 | 5140 |  |
| Demographics | | | | | | |
| Age*, years* | 73.0 [69.0-77.0] | 81.0 [76.0-84.0] | **0.001*** | 75.0 [71.0-79.0] | 82.0 [77.0-85.0] | **<0.001*** |
| Sex *(Female)* | 3056 (44.5) | 4921 (52.8) | **<0.001*** | 391 (38.6) | 1311 (45.0) | **<0.001*** |
| BMI | 27.3 [24.7-30.4] | 26.7 [24.1-29.9] | **<0.001*** | 27.6 [24.4-30.8] | 26.1 [23.6-29.6] | **<0.001*** |
| NYHA Class III/IV | 1618 (30.6) | 4686 (53.8) | **<0.001*** | 276 (40.9) | 3026 (64.6) | **<0.001*** |
| CCS Class IV | 49 (0.83) | 186 (2.22) | **<0.001*** | 12 (1.50) | 116 (2.69) | 0.062 |
| Poor Mobility | 178 (3.07) | 726 (9.08) | **<0.001*** | 38 (4.90) | 455 (11.5) | **<0.001*** |
| EuroSCORE II | 1.40 [1.06-2.00] | 2.98 [1.91-4.80] | **0.001*** | 1.89 [1.31-3.22] | 4.00 [2.38-6.88] | **<0.001*** |
| Comorbidities | | | | | | |
| Chronic Lung Disease | 786 (11.5) | 1544 (16.6) | **<0.001*** | 209 (20.7) | 1274 (24.9) | **0.005*** |
| Diabetes | 1362 (20.1) | 2284 (24.7) | **<0.001*** | 308 (31.0) | 1605 (31.8) | 0.640 |
| Atrial Fibrillation | 465 (10.4) | 478 (28.1) | **<0.001*** | 100 (17.2) | 130 (42.2) | **<0.001*** |
| Dialysis | 12 (0.20) | 45 (0.49) | **0.008*** | 10 (1.21) | 84 (1.67) | 0.408 |
| Stroke | 291 (4.55) | 887 (9.52) | **<0.001*** | 69 (7.25) | 623 (12.1) | **<0.001*** |
| Cardiac Status | | | | | | |
| Unstable Angina | 10 (0.15) | 33 (0.36) | **0.016*** | 1 (0.10) | 11 (0.22) | 0.704 |
| Recent MI | 73 (1.06) | 171 (1.84) | **<0.001*** | 14 (1.39) | 104 (2.04) | 0.207 |
| Previous Cardiac Surg*.* | 238 (3.47) | 1378 (14.9) | **<0.001*** | 64 (6.32) | 837 (16.7) | **<0.001*** |
| Thoracic Aortic Surg*.* | 3 (0.04) | 2 (0.02) | 0.657 | 0 (0.00) | 3 (0.06) | 1.000 |
| Endocarditis | 169 (2.46) | 0 (0.00) | **<0.001*** | 43 (4.25) | 2 (0.04) | **<0.001*** |
| Critical Pre-op. Cond. | 51 (0.74) | 26 (0.28) | **<0.001*** | 19 (1.88) | 31 (0.61) | **<0.001*** |
| Urgency | 786 (11.9) | 717 (7.78) | **<0.001*** | 158 (16.7) | 577 (11.5) | **<0.001*** |
| Laboratory values | | | | | | |
| Creatinine *(μmol/l)* | 82.0 [70.0-96.0] | 88.0 [73.0-108] | **<0.001*** | 87.0 [73.0-108] | 99.0 [80.0-129] | **<0.001*** |
| Echocardiography | | | | | | |
| LVEF | 55.0 [55.0-56.0] | 55.0 [45.0-55.0] | **<0.001*** | 55.0 [50.5-56.0] | 55.0 [40.0-55.0] | **<0.001*** |
| PA-Pressure *(mmHg)* | 25.0 [25.0-25.0] | 25.0 [25.0-29.0] | **<0.001*** | 25.0 [25.0-25.0] | 25.0 [25.0-35.0] | **<0.001*** |
| Data are presented as n (%) or median [interquartile range].  * P value of <0.05 is considered statistically significant.  BMI: Body Mass Index; CCS: Canadian Cardiovascular Society Classification; EuroSCORE: European System for Cardiac Operative Risk Evaluation; LVEF: Left Ventricular Ejection Fraction; MI: Myocardial Infarction; NYHA: New York Heart Association Functional Classification; PASP: pulmonary arterial systolic pressure; SAVR: Surgical Aortic Valve Replacement; TAVI: Transcatheter Aortic Valve Implantation | | | | | | |
