# Supplementary material for: Long-term age-stratified outcomes after surgical and transcatheter aortic valve replacement: a Dutch cohort study
Source: Neth Heart J. 2025 Apr 11;33(5):172–9. doi: 10.1007/s12471-025-01944-5 (PMC12014882; doi:10.1007/s12471-025-01944-5)
Supplement: Supplementary file 8 — Table S8 Outcomes per age-group, stratified by cohort [file 12471_2025_1944_MOESM8_ESM.docx]

**Table S8** Outcomes per age-group- stratified by cohort

| Age-group | 65-75 years | | | | 75-80 years | | | | >80 years | | |
| --- | --- | --- | --- | --- | --- | --- | --- | --- | --- | --- | --- |
| Cohort | **SAVR** | **TAVI** |  | **SAVR** | | **TAVI** |  | **SAVR** | | **TAVI** |  |
| N | **N=5428** | **N=2715** | **p** | **N=2708** | | **N=4658** | **p** | **N=799** | | **N=8553** | **p** |
| Mortality n(%) | 467 (9.7) | 811 (32.8) | **<0.001*** | 374 (15.7) | | 1354 (32.1) | **<0.001*** | 171 (24.5) | | 2975 (38.3) | **<0.001*** |
| Time to mortality  (median [IQR]) | 890.0  [356.5- 1345.5] | 608.0  [206.5- 1097.0] | **<0.001*** | 827.5  [288.5- 1339.8] | | 648.0  [252.8- 1135.0] | **0.001*** | 946.0  [321.5- 1377.5] | | 721.0  [265.5- 1192.5] | **0.002*** |
| *1-year mortality n(%) | 120 (2.5) | 285 (11.5) | **<0.001*** | 114 (4.8) | | 423 (10.0) | **<0.001*** | 49 (7.0) | | 907 (11.7) | **<0.001*** |
| Aortic valve re-intervention n(%) | 101 (2.4) | 32 (1.5) | **0.020*** | 29 (1.4) | | 34 (0.9) | 0.097 | 3 (0.5) | | 46 (0.7) | 0.878 |
| Time to re-intervention  (median [IQR]) | 401.0  [129.0- 1006.0] | 125.0  [25.2- 592.0] | **0.010*** | 181.0  [55.0- 458.0] | | 58.5  [12.5- 234.2] | 0.069 | 3.0  [1.5- 174.0] | | 34.5  [9.8- 135.2] | 0.427 |
| 1-year re-intervention n(%) | 47 (1.1) | 22 (1.0) | 0.826 | 20 (1.0) | | 27 (0.7) | 0.372 | 3 (0.5) | | 39 (0.6) | 1.000 |
| Days of follow up  (median [IQR]) | 1611.0  [1087.0- 1794.0] | 1121.0  [618.8- 1613.0] | **<0.001*** | 1614.0  [1056.0- 1785.0] | | 1126.5  [646.0- 1604.8] | **<0.001*** | 1662.0  [1251.0- 1806.0] | | 1120.0  [622.0- 1598.0] | **<0.001*** |
| * P value of <0.05 is considered statistically significant  Time to event by log-rank test. | | | | | | | | | | | |
