# Supplementary material for: Long-term age-stratified outcomes after surgical and transcatheter aortic valve replacement: a Dutch cohort study
Source: Neth Heart J. 2025 Apr 11;33(5):172–9. doi: 10.1007/s12471-025-01944-5 (PMC12014882; doi:10.1007/s12471-025-01944-5)
Supplement: Supplementary file 9 — Table S9 Mortality per 100 patient years [file 12471_2025_1944_MOESM9_ESM.docx]

**Table S9** Mortality per 100 patient years

| Age Group | Cohort | Total Events | Total event time  (days) | Events (95% CI)  (per 100 patient years) |
| --- | --- | --- | --- | --- |
| 65-75 | SAVR | 467 | 18469 | 2.53 (2.30-2.77) |
|  | TAVI | 811 | 7332 | 11.06 (10.31-11.85) |
| 75-80 | SAVR | 374 | 9010 | 4.15 (3.74-4.59) |
|  | TAVI | 1354 | 12649 | 10.70 (10.14-11.29) |
| >80 | SAVR | 171 | 2753 | 6.21 (5.32-7.22) |
|  | TAVI | 2975 | 22879 | 13.00 (12.54-13.48) |
| Events are defined as all-casue mortality.  * P value of <0.05 is considered statistically significant  Time to event by log-rank test. | | | | |
