# Supplementary material for: Long-term age-stratified outcomes after surgical and transcatheter aortic valve replacement: a Dutch cohort study
Source: Neth Heart J. 2025 Apr 11;33(5):172–9. doi: 10.1007/s12471-025-01944-5 (PMC12014882; doi:10.1007/s12471-025-01944-5)
Supplement: Supplementary file 10 — Table S10 Hazard ratios of cohort and age-group [file 12471_2025_1944_MOESM10_ESM.docx]

**Table S10** Hazard ratios of cohort and age-group

|  | HR (95% CI) | p-value | p-interaction^†^ |
| --- | --- | --- | --- |
| TAVI  (versus SAVR, in 65-75 age-group) | 4.47 (3.98-5.01) | **<0.001** | **<0.001** |
| TAVI x age-group 75-80 ^†^ | 0.60 (0.51-0.70) | **<0.001** |  |
| TAVI x age-group >80 ^†^ | 0.50 (0.41-0.60) | **<0.001** |  |
| ^†^Hazard ratio of TAVI and age group versus reference of SAVR, interaction term with age-group.  Analysis shows stratified age-groups because of non-proportional hazards for age-group (by Schoenfeld Residuals) | | | |
